# Complete and Durable Response to Tamoxifen and Ovarian Ablation in the Treatment of Metastatic Breast Cancer

**DOI:** 10.7759/cureus.14088

**Published:** 2021-03-24

**Authors:** Hikmat Abdel-Razeq, Mahmoud Abunasser, Heba Farfoura, Rshid Abdel-Razeq, Jakub Khzouz

**Affiliations:** 1 Medical Oncology, King Hussein Cancer Center, Amman, JOR; 2 Medicine, The University of Jordan, Amman, JOR; 3 Radiology, King Hussein Cancer Center, Amman, JOR; 4 Internal Medicine, Istishari Hospital, Amman, JOR; 5 Pathology and Laboratory Medicine, King Hussein Cancer Center, Amman, JOR

**Keywords:** hormone receptor-positive breast cancer, hormone therapy, tamoxifen, cdk4/6 inhibitor, metastasis

## Abstract

Most breast cancers are hormone receptor-positive, and tamoxifen is the mainstay of endocrine therapy for such patients. However, with the introduction of aromatase inhibitors and then fulvestrant, tamoxifen became a less preferred drug, even in a premenopausal setting. Many new approaches to tackle the recently identified resistance pathways for endocrine therapy are widely used. Cyclin-dependent kinase 4/6 inhibitors such as ribociclib, palbociclib, and abemaciclib, and inhibitors of the mechanistic target of rapamycin such as everolimus, are effective but have been associated with considerably higher costs and distinctive toxicities.

We describe the clinical course of a young patient with advanced-stage breast cancer living in an under-resourced region who failed to respond to chemotherapy, including both anthracyclines and taxanes, but achieved complete and durable response to tamoxifen and ovarian ablation therapy.

This case demonstrates the value of well-established, low-cost endocrine therapy for breast cancer. It also highlights the need to address the sequencing of endocrine therapy for patients in low-income countries.

## Introduction

For over 100 years, hormonal manipulation to treat breast cancer has been applied. In 1896, Sir George Thomas Beatson showed that oophorectomy resulted in tumor regression for unresectable breast cancer [1]. Since then, estrogen receptor (ER) modulators and estrogen deprivation have been widely used to treat hormone receptor (HR)-positive metastatic breast cancer (mBC) [2]. Tamoxifen, the most widely used hormonal therapy for breast cancer, was first synthesized in 1962 with the initial aim of being a morning-after contraceptive pill. Despite extensive research, this aim was not achieved and efforts were then directed towards understanding its anti-tumor activity. The first clinical study to demonstrate a convincing anti-cancer effect of tamoxifen in advanced breast cancer was conducted at the Christie Hospital in 1971 [3], and the results of that study were then supported by a United Kingdom study undertaken at the Queen Elizabeth Hospital in Birmingham [4]. Subsequently, the drug emerged as the gold standard endocrine therapy (ET) for patients with breast cancer.

In a 2012 report involving 20,000 women in 20 randomized trials, the Early Breast Cancer Trialists’ Collaborative Group reported that five years of tamoxifen versus no tamoxifen resulted in a highly significant one-third reduction in breast cancer mortality [5].

Many breast cancer patients are sensitive and highly responsive to tamoxifen. Nevertheless, recent advances in molecular biology that have led to the identification of multiple resistance pathways, and the recent introduction of many new drugs to tackle such pathways, have led to the underutilization of this simple and potent drug.

Several recent prospective randomized clinical trials have demonstrated that the addition of agents that work in ways other than inducing ER interference or estrogen depletion can enhance the clinical benefit of ET. In particular, cyclin-dependent kinase (CDK) 4/6 inhibitors in combination with ET are currently used in first-line therapy for patients with mBC. Three agents in this group, namely, palbociclib [6], ribociclib [7], and abemaciclib [8] improve progression-free survival (PFS) when added to ET as first-line or subsequent therapies. Ribociclib was the first among the three to demonstrate a survival advantage in both premenopausal and postmenopausal settings [9,10].

Everolimus, an inhibitor of the mechanistic target rapamycin, has also demonstrated improved PFS when added to ET in the endocrine-resistant setting [11]. Currently, there have been no randomized trials to compare everolimus with various CDK 4/6 inhibitors in patients receiving ET.

Although such agents have clearly shown an added value in terms of response rates, PFS, and overall survival, the tremendous increase in cost remains the major issue concerning these agents, particularly in low-income countries.

## Case presentation

A 30-year-old female patient from a less-developed region was first seen at our institution almost seven years ago for evaluation of mBC. This patient had been diagnosed a few months earlier at another institution with infiltrating ductal carcinoma (mucinous type). Both ER and progesterone (PR) receptors were positive in 90% and 60% of the cells, respectively, while Human Epidermal Growth Factor Receptor (HER/neu) was negative on immunohistochemical staining. Her pathology results were reviewed and confirmed at our institution.

**Figure 1:**
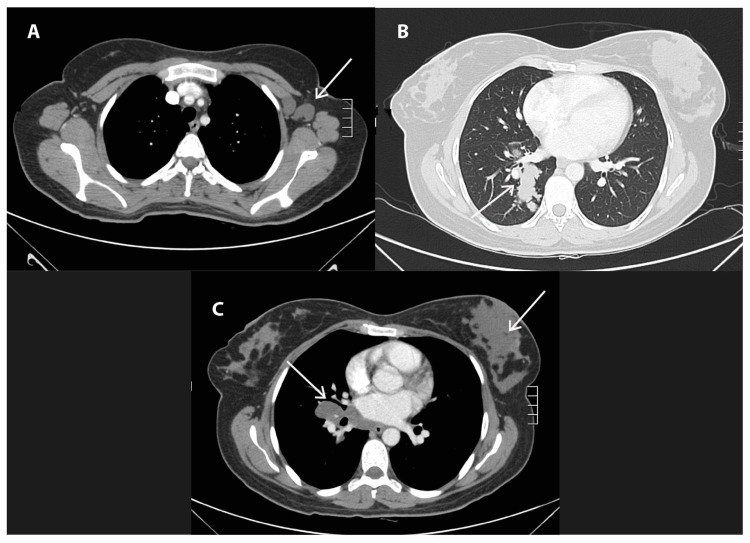
Imaging studies of the tumor prior to endocrine therapy. (A) Axillary lymph node (arrows) 2.4 x 1.8 cm. (B) Lung mass (arrow). (C) Hilar lymph node (left arrow) and breast.

Staging at the time of diagnosis and at presentation to our institution showed a tumor in the left breast and left axillary lymph node involvement, in addition to right hilar lymph node involvement and a right lung mass (Figure 1).

**Figure 2:**
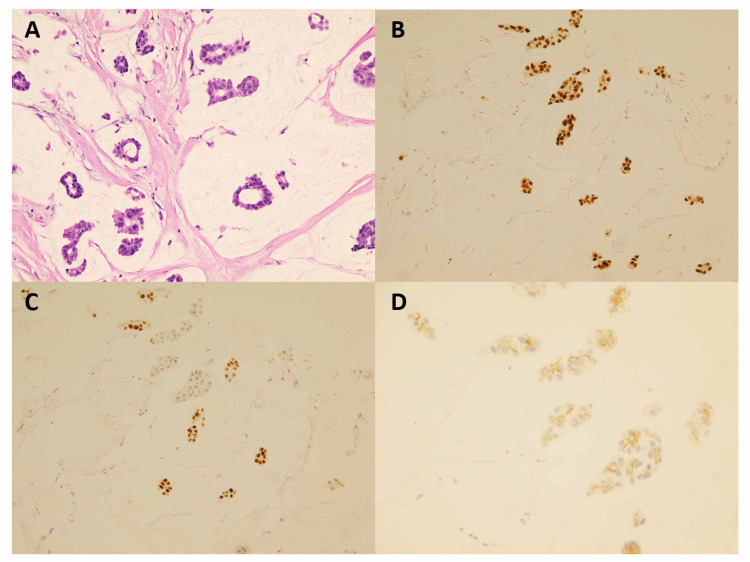
Pathology of resected tumor. (A) Mucinous/colloid mammary carcinoma showing malignant cells arranged in ducts embedded in pools of extracellular mucinous material (hematoxylin-eosin, original magnification x20). (B) Most of the tumor cells are positive for estrogen receptors immunostain (ER [SP1] immunostain, original magnification x20). (C) Tumor cells are positive for progesterone receptors immunostain (PR [1E2] immunostain, original magnification x20). (D) Tumor cells are negative for Her-2/neu (score 1+0 Her-2/neu Rabbit monoclonal [4B5] immunostain, original magnification x40).

She was treated with four cycles of adriamycin and cyclophosphamide; however, there was no significant response. She was then switched to docetaxel and four cycles were administered, again with no response. Given the lack of response, both hilar lymph nodes and the lung mass were biopsied and pathology test results confirmed the original diagnosis of invasive mucinous carcinoma (Figure 2). Again, both ER and PR were strongly positive while HER2/new was negative.

**Figure 3:**
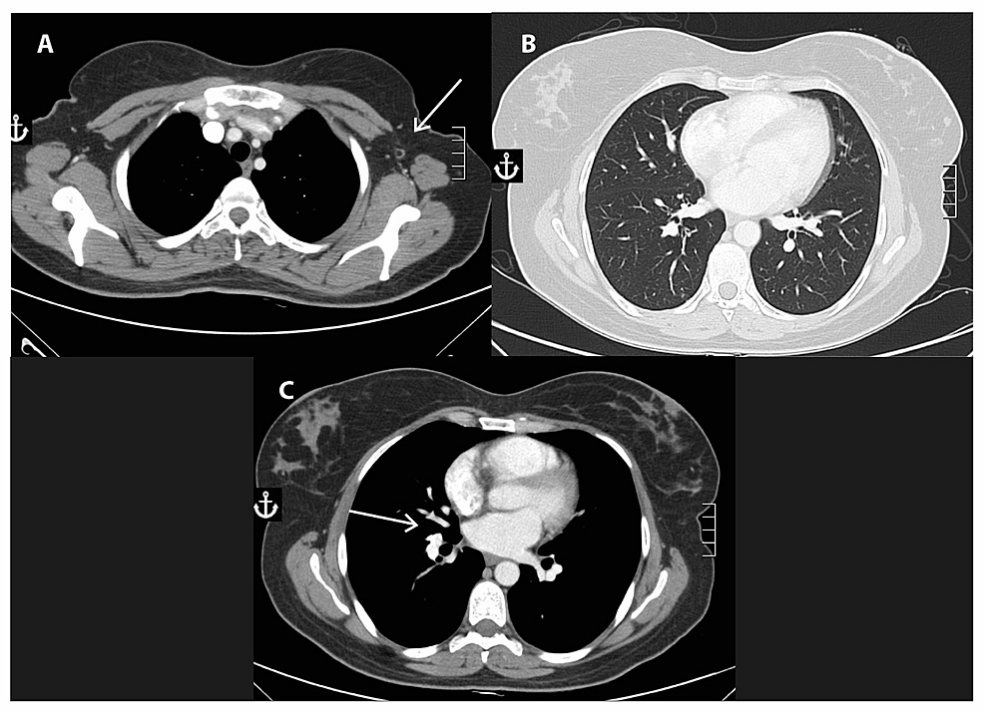
Follow-up imaging studies. Imaging studies of the tumor undertaken nine months after endocrine therapy showing disappearance of the affected axillary lymph nodes (A), disappearance of the lung mass (B), and disappearance of the hilar lymph node (C).

Given the stable nature of her disease, the absence of a visceral crisis, and because of the patient’s social issues and the limited availability of appropriate medication in her home region, she was started on tamoxifen along with ovarian ablation using gonadotropin-releasing hormone (GnRH) agonists (goserelin, 3.6 mg subcutaneously, every four weeks). Follow-up imaging studies showed a significant disease response three and six months later. Our patient continued on the same treatment, with no major side effects. Follow-up CT scans at nine months showed a continued response in the breast mass and complete resolution of the right lung mass and the right hilar lymph nodes. Moreover, tumors in the left axillary lymph nodes had completely disappeared (Figure 3).

Given her remarkable response, she underwent a mastectomy, the pathology of which showed residual multifocal mucinous adenocarcinoma with at least 3 masses identified, the largest being 2.5 cm in size. Four axillary lymph nodes were examined and were found to be negative but with treatment effect. Following the surgery, she underwent radiotherapy (50 Gy in 25 fractions) to the left chest wall and supraclavicular area. Tamoxifen and GnRH agonist were continued with regular follow-up both clinically and using imaging studies. The most recent follow-up was undertaken seven years after initiation of tamoxifen and GnRH agonist treatment and her status remains disease-negative.

Our patient provided written informed consent to publish her case report, including consent to publish the results of her pathology tests and radiology images.

## Discussion

Given the lack of predictive biomarkers, it is not possible to accurately predict which patients are likely to achieve such an extraordinary benefit from simple ET. However, given the complicated socioeconomic issues associated with our patient and the indolent course of her illness, we opted to proceed with a low-cost regimen. The use of higher cost ET regimens such as AI, fulvestrant, or a combination of everolimus and AI was prohibitively expensive and not feasible for this particular patient. Moreover, no CDK 4/6 inhibitors were available at the time.

The duration of remission obtained in our patient using tamoxifen and GnRH agonist was clearly considerably longer than has been achieved using more-recently introduced ET options. The combination of CDK4/6 inhibitors plus AI has been reported to have a PFS of 24.8 months with palbociclib [6], 25.3 months with ribociclib [7], and 28.2 months with abemaciclib [8,12]. Fulvestrant monotherapy was found to have a 16.6-month PFS in the Falcon trial [13] and no significant addition when combined with AI [14,15].

## Conclusions

Our case clearly illustrates that even in the era of sophisticated ET regimens such as CDK 4/6 inhibitors along with AI or fulvestrant, simple regimens such as tamoxifen can still be effective especially when the disease course is indolent, and when the tumor load is not considerable and does not involve vital structures. Our case report also highlights the importance of ET sequencing; given the chronic nature of HR-positive mBC, particular sequencing might not be a factor that affects overall survival.
